# Supplementary material for: Experimental Comparative Study of Dynamic Behavior in Solution Phase of C-Tetra(phenyl)resorcin[4]arene and C-Tetra(phenyl)pyrogallol[4]arene
Source: Molecules. 2020 May 12;25(10):2275. doi: 10.3390/molecules25102275 (PMC7287697; doi:10.3390/molecules25102275)
Supplement: Supplementary file 1 [file molecules-25-02275-s001.pdf]

Experimental Comparative study of dynamic behavior in solution phase of *C*-tetra(phenyl)resorcin[4]arene and C-tetra(phenyl)pyrogallol[4]arene

José Luis Casas-Hinestroza, Miguel Ángel Vela Suazo and Mauricio Maldonado

Table of contents:

Figure S1. ^{1}H-NMR spectrum DMSO-d_6 conformational mixture(**1a** and **1b**)

Figure S2. IR Spectrum of tetra(phenyl)-resorcin[4]arene(**1a**)

Figure S3. ^{1}H-NMR spectrum-DMSO-d_6 of tetra(phenyl)-resorcin[4]arene(**1a**)

Figure S4. ^{13}C-NMR spectrum- DMSO-d_6 of tetra(phenyl)-resorcin[4]arene*(*1a)

Figure S5. 2D-NMR-HSQC spectrum-DMSO-d_6 of tetra(phenyl)-resorcin[4]arene(**1a**)

Figure S6. Dynamic ^{1}H-NMR study for tetra(phenyl)-resorcin[4]arene(**1a**)

Figure S7. IR Spectrum of tetra(phenyl)-resorcin[4]arene(**1b**)

Figure S8. ^{1}H-NMR spectrum-DMSO-d_6 of tetra(phenyl)-resorcin[4]arene(**1b**)

Figure S9. ^{13}C-NMR spectrum of tetra(phenyl)-resorcin[4]arene (**1b**)

Figure S10. ^{1}H-NMR spectra of boat(*rccc*)**(1a*)*** and chair(*rctt*)**(1b)** isomers

Figure S11. ^{1}H-NMR spectrum DMSO-d_6 conformational mixture(**2a** and **2b**)

Figure S12. IR Spectrum of tetra(phenyl)-pyrogallol[4]arene(**2a**)

Figure S13. ^{1}H-NMR spectrum- DMSO-d_6 of tetra(phenyl)-pyrogallol[4]arene(**2a**)

Figure S14. ^{13}C-NMR spectrum- DMSO-d_6 of tetra(phenyl)-pyrogallol[4]arene(**2a)**

Figure S15. Dynamic ^{1}H-NMR study for tetra(phenyl)-pyrogallol[4]arene(**2a**)

Figure S16. IR Spectrum of tetra(phenyl)-pyrogallol[4]arene(**2a**)

Figure S17. ^{1}H-NMR spectrum-DMSO-d_6 of tetra(phenyl)-pyrogallol[4]arene(**2b**)

Figure S18. ^{13}C-NMR spectrum- DMSO-d_6 of tetra(phenyl)-pyrogallol[4]arene(**2b**)

Figure S19. ^{1}H-NMR spectra of conformational mixture, *boat*(*rccc*)(**2a**) and *chair*(*rctt*)(**2b**) isomers

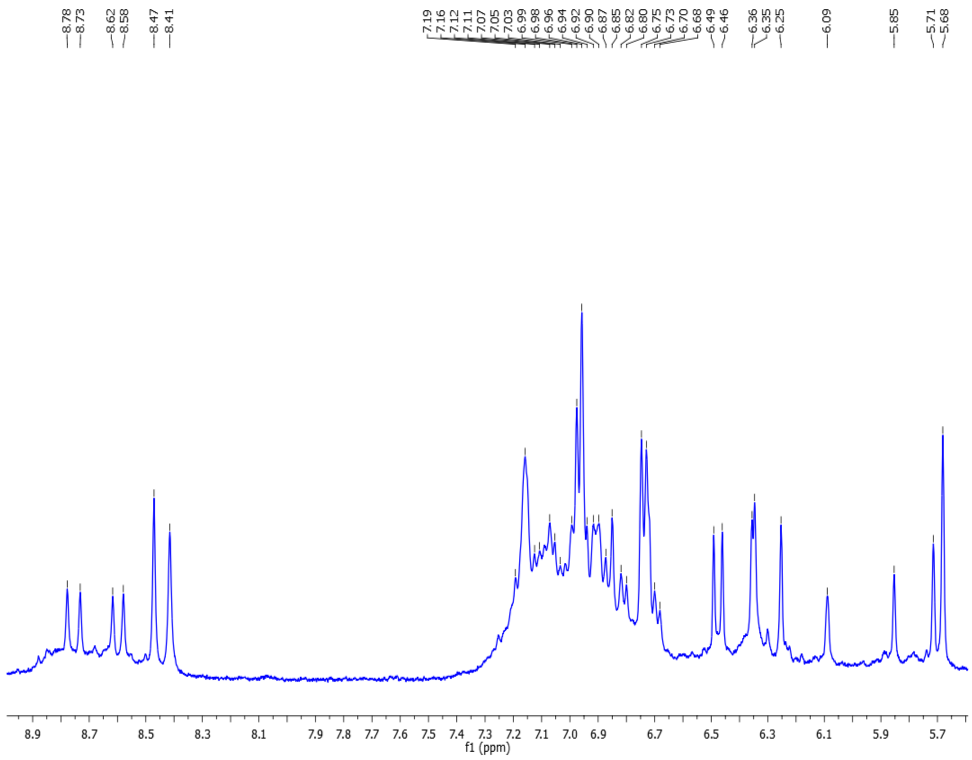

Figure S1. ^{1}H-NMR spectrum DMSO-*d₆* conformational mixture(**1a** and **1b**)

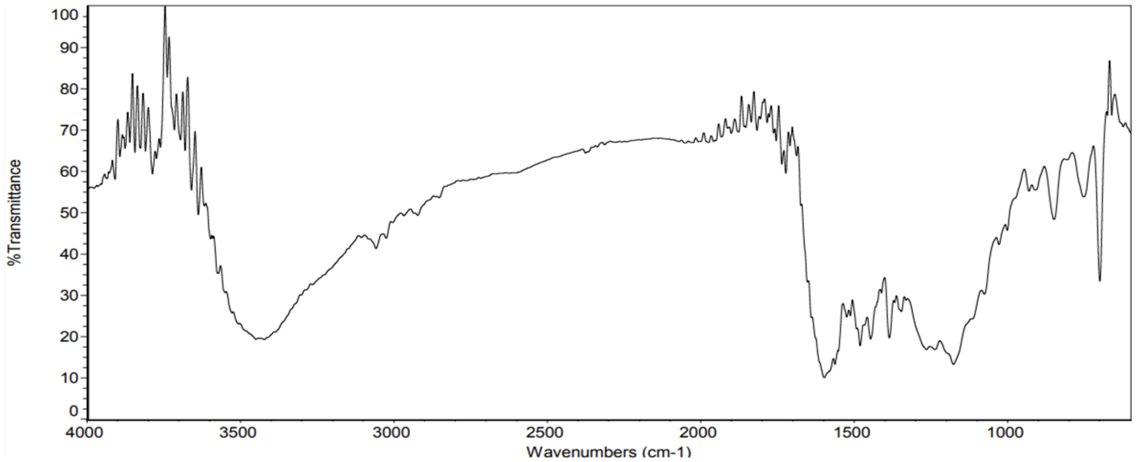

Figure S2. IR Spectrum of tetra(phenyl)-resorcin[4]arene(**1a**)

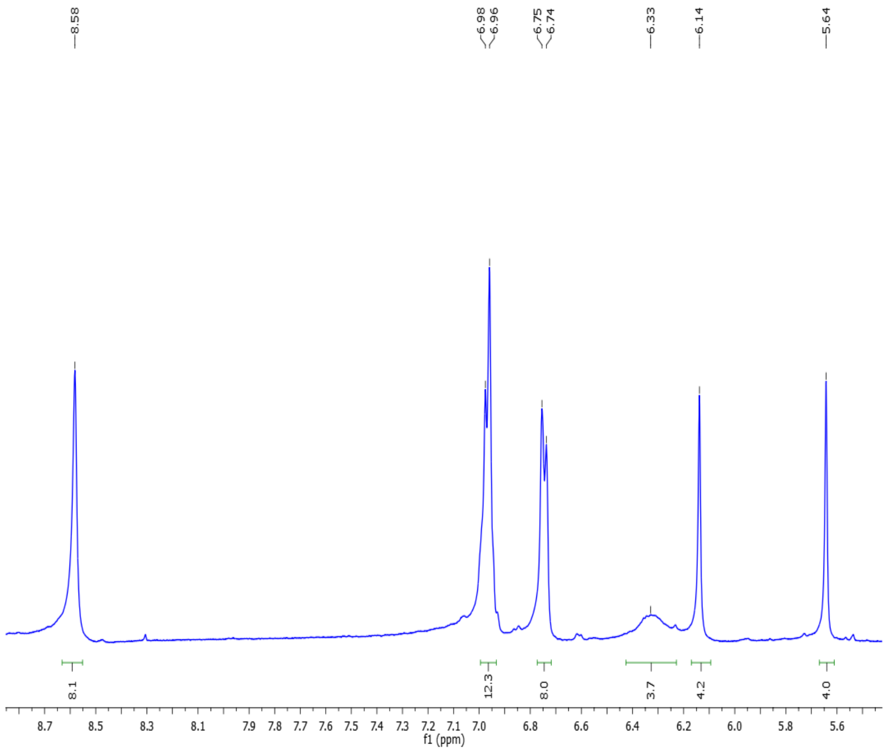

Figure S3. ^{1}H-NMR spectrum-DMSO-*d*6 of tetra(phenyl)-resorcin[4]arene(**1a**)

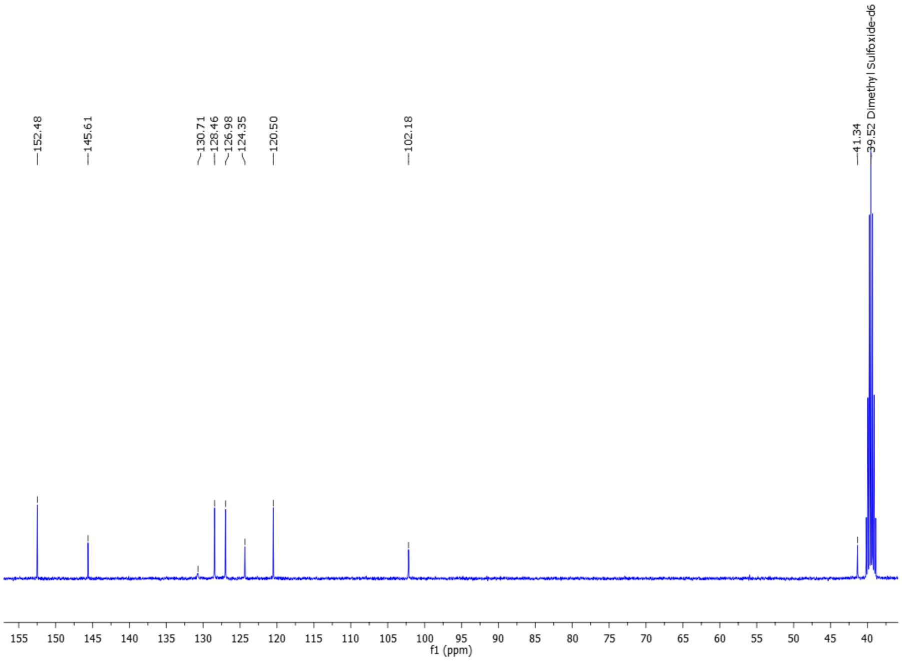

Figure S4. ^{13}C-NMR spectrum- DMSO-*d*6 of tetra(phenyl)-resorcin[4]arene(**1a**)

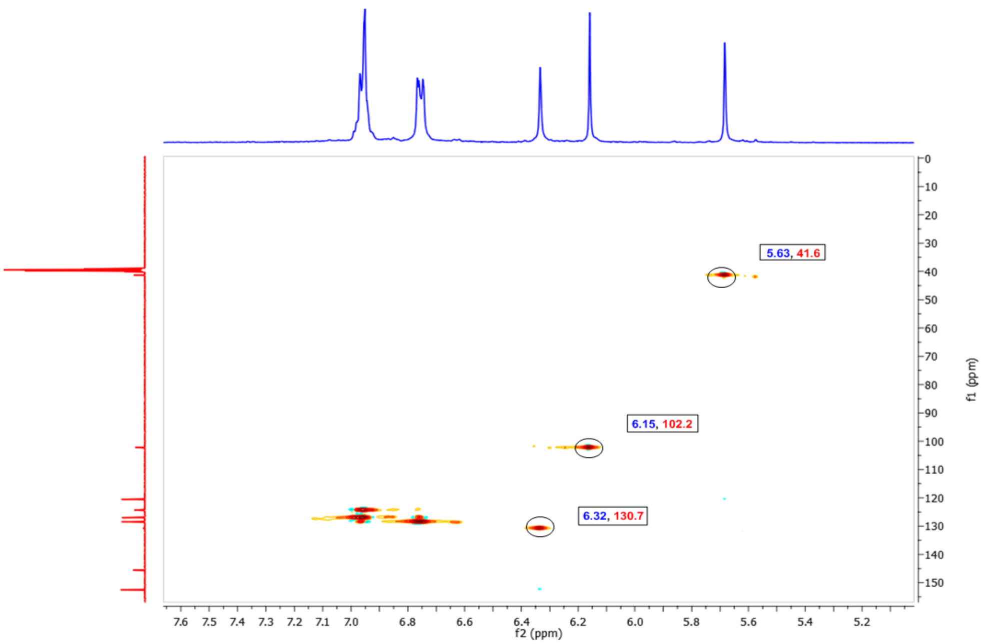

Figure S5. 2D-NMR-HSQC spectrum-DMSO-d_6 of tetra(phenyl)-resorcin[4]arene(**1a**)

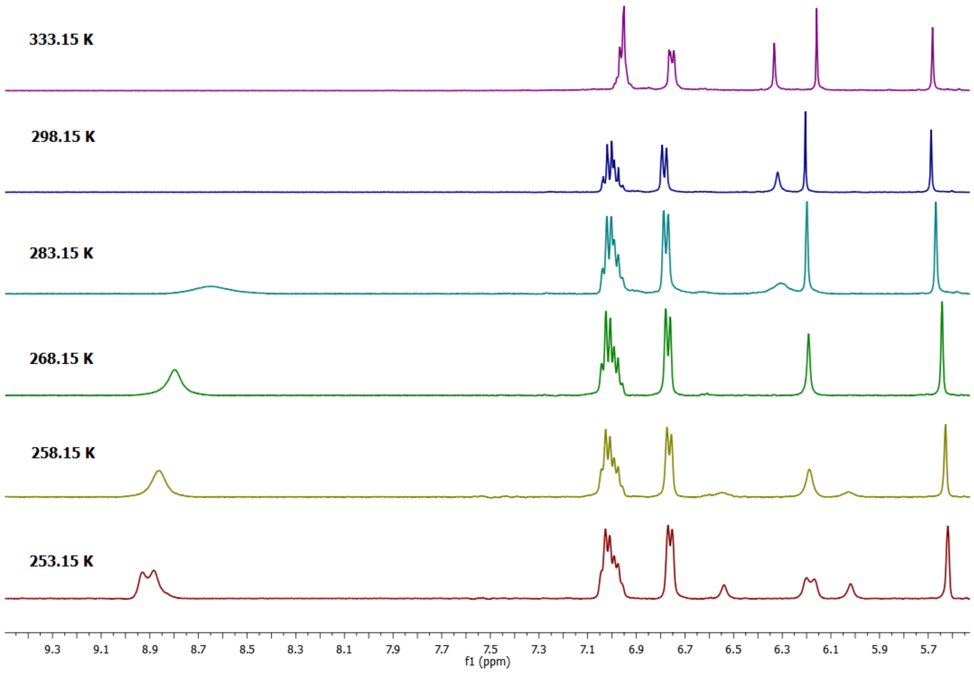

Figure S6. Dynamic ^{1}H-NMR study for tetra(phenyl)-resorcin[4]arene(**1a**)

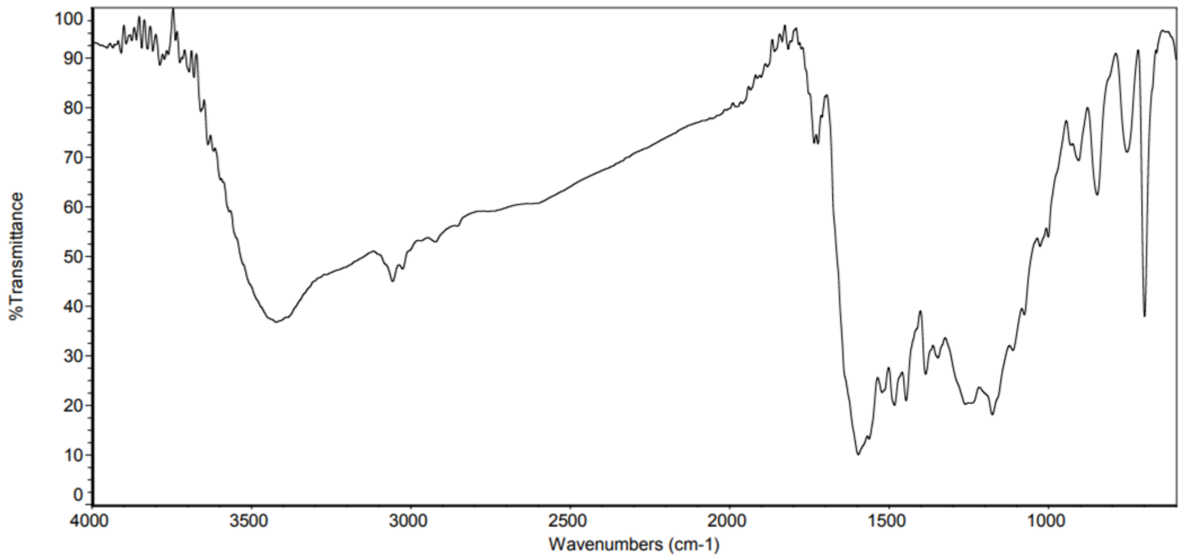

Figure S7. IR Spectrum of tetra(phenyl)-resorcin[4]arene(**1b**)

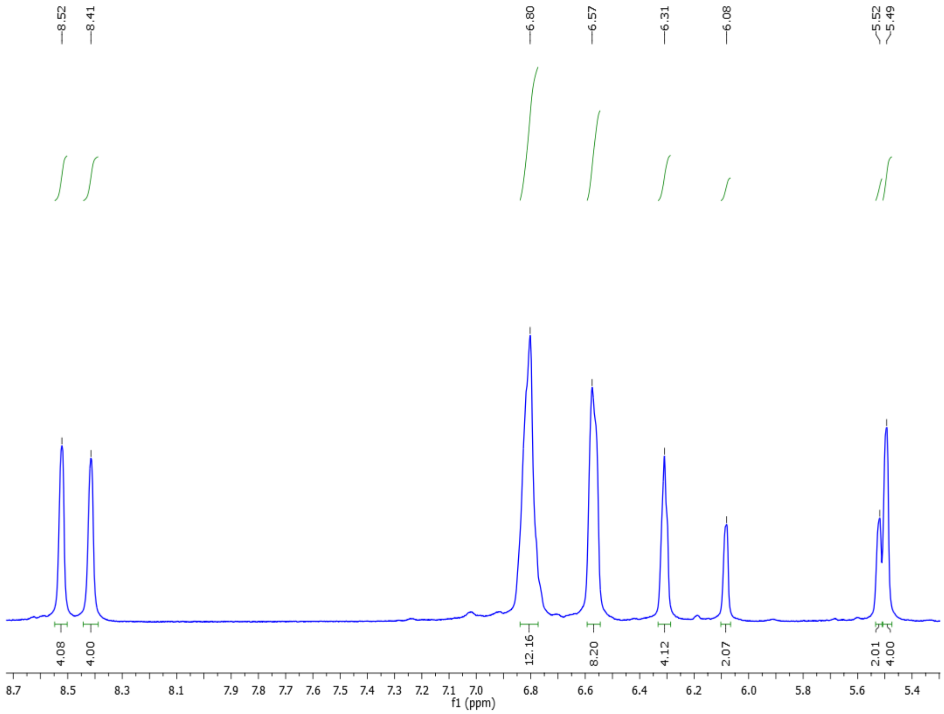

Figure S8. ^{1}H-NMR spectrum-DMSO-d_6 of tetra(phenyl)-resorcin[4]arene(**1b**)

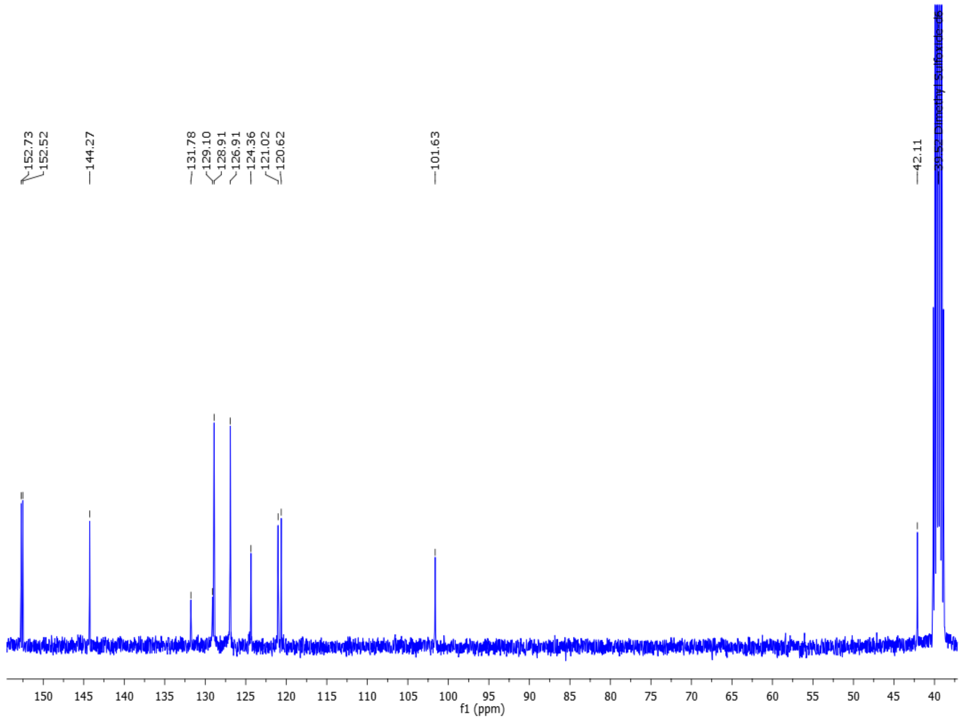

Figure S9. ^{13}C-NMR spectrum- DMSO-d_6 of tetra(phenyl)-resorcin[4]arene(**1b**)

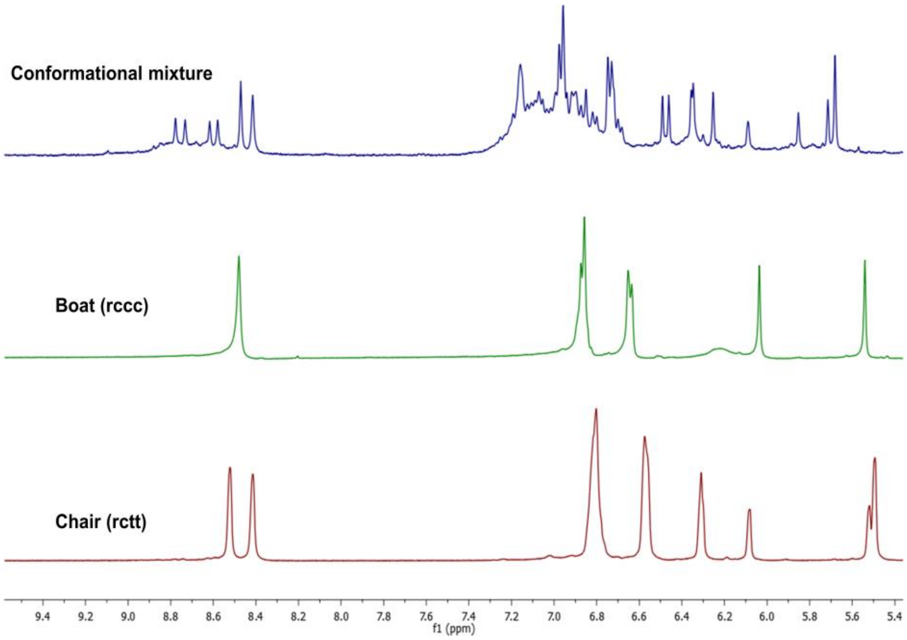

Figure S10. ^{1}H-NMR spectra of *boat*(*rccc*)(**1a**) and *chair*(*rctt*)(**1b**) isomers

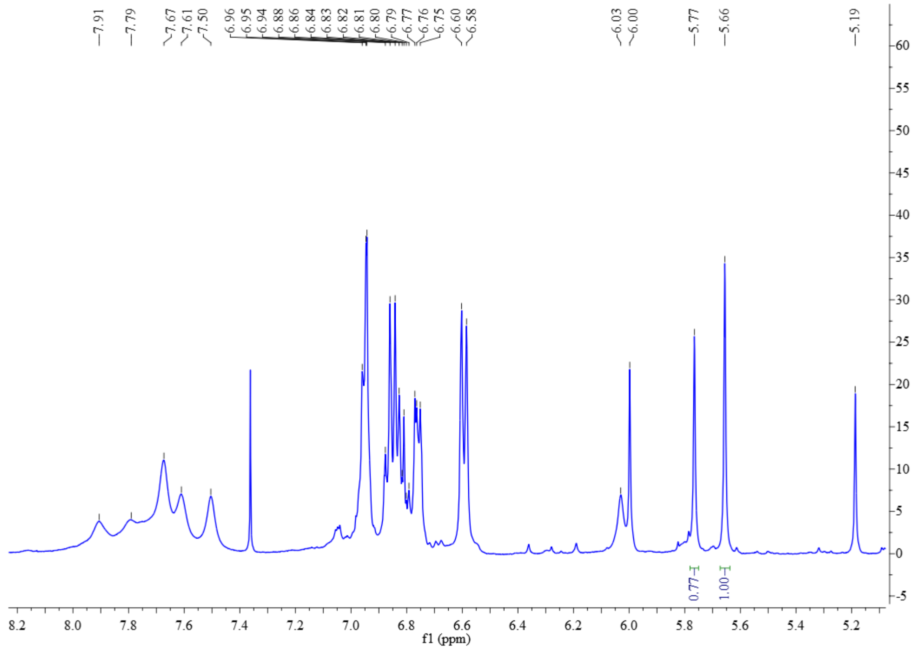

Figure S11. ^{1}H-NMR spectrum DMSO-d_6 conformational mixture(**2a** and **2b**)

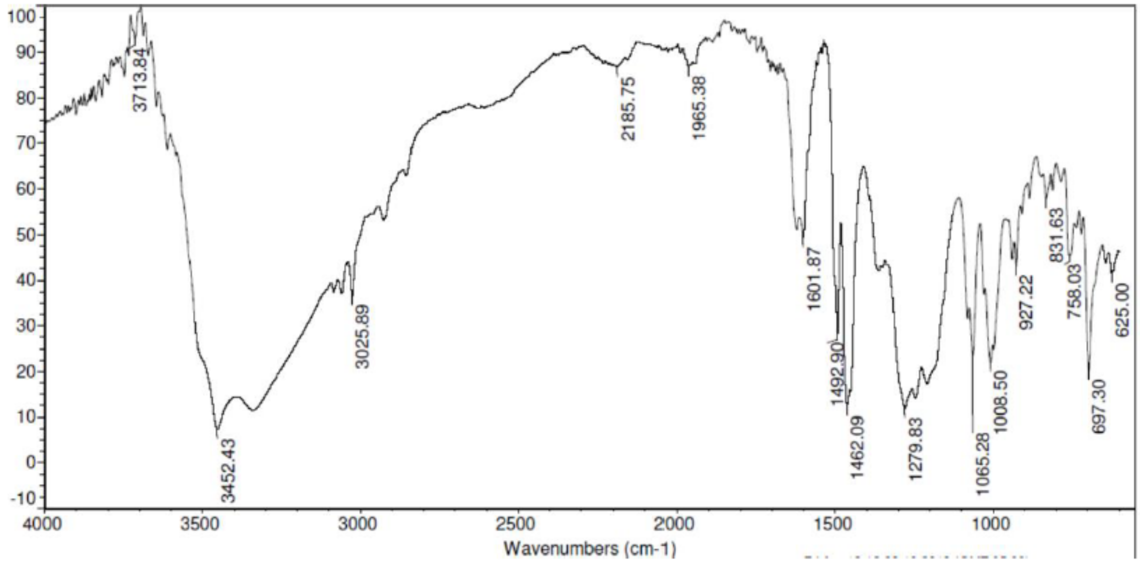

Figure S12. IR Spectrum of tetra(phenyl)-pyrogallol[4]arene(**2a**)

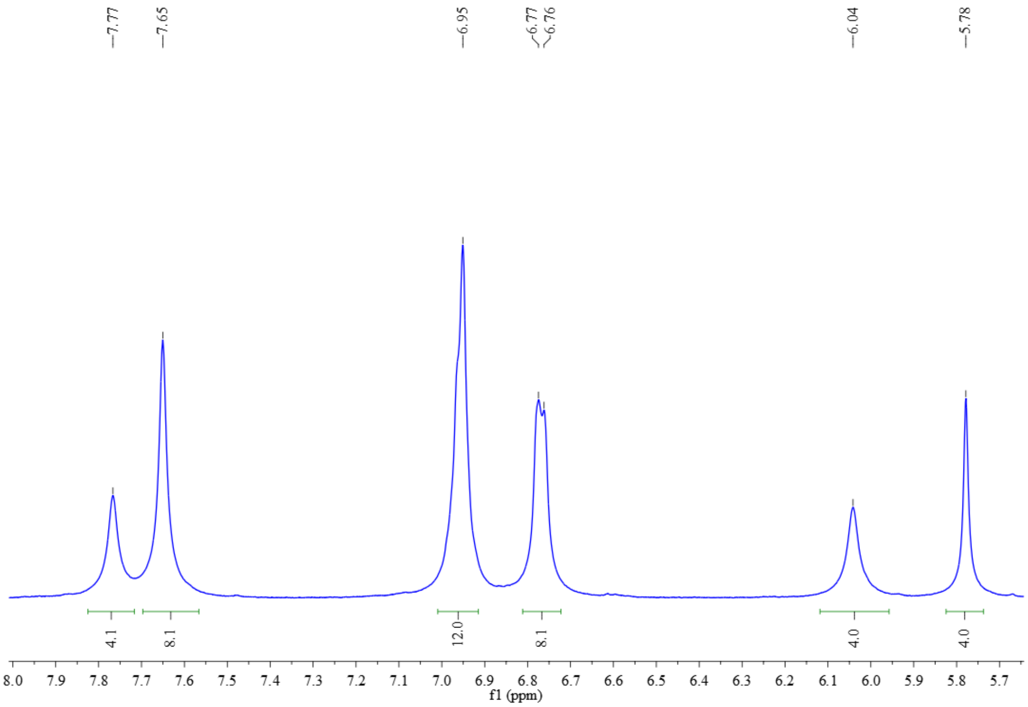

Figure S13. ^{1}H-NMR spectrum- DMSO-d_6 of tetra(phenyl)-pyrogallol[4]arene(**2a**)

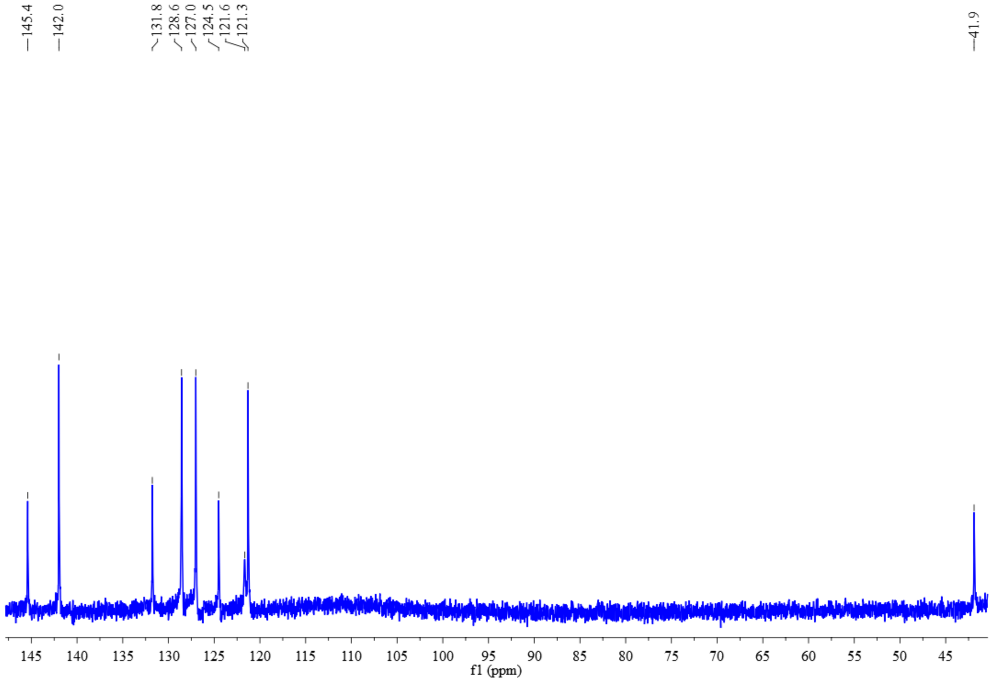

Figure S14. ^{13}C-NMR spectrum- DMSO-d_6 of tetra(phenyl)-pyrogallol[4]arene(**2a)**

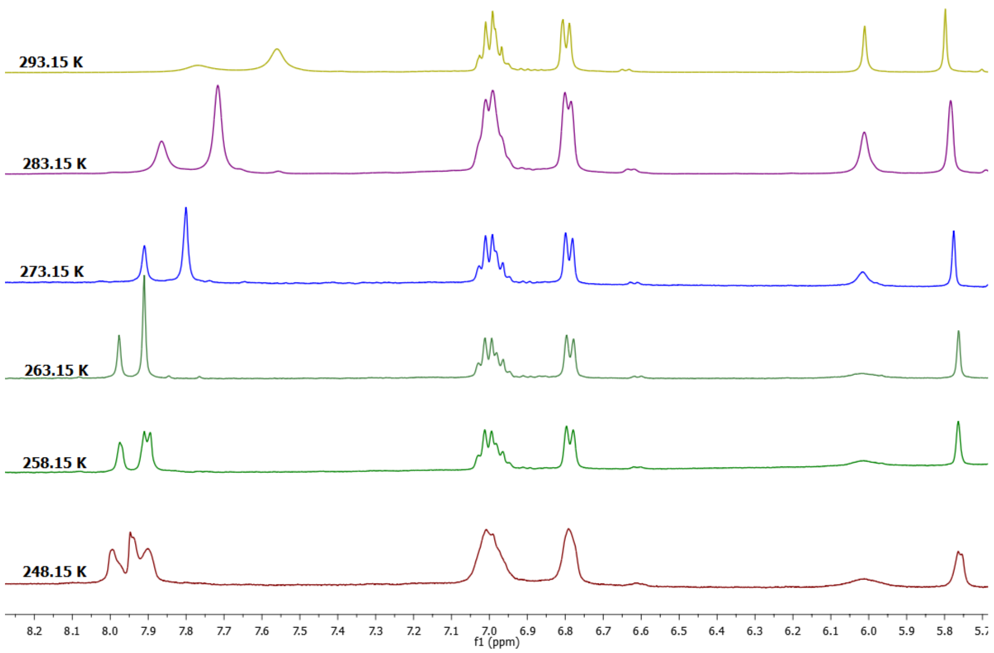

Figure S15. Dynamic ^{1}H-NMR study for tetra(phenyl)-pyrogallol[4]arene(**2a**)

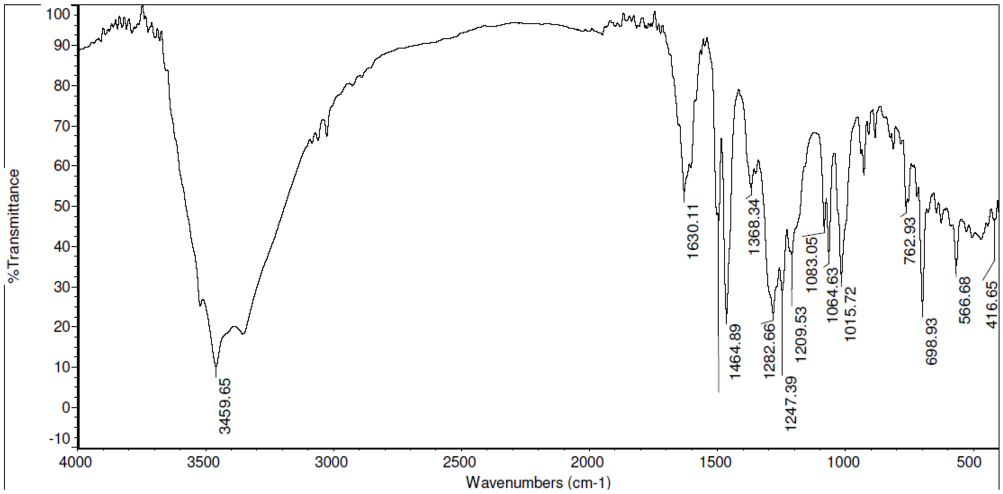

Figure S16. IR Spectrum of tetra(phenyl)-pyrogallol[4]arene (**2b**)

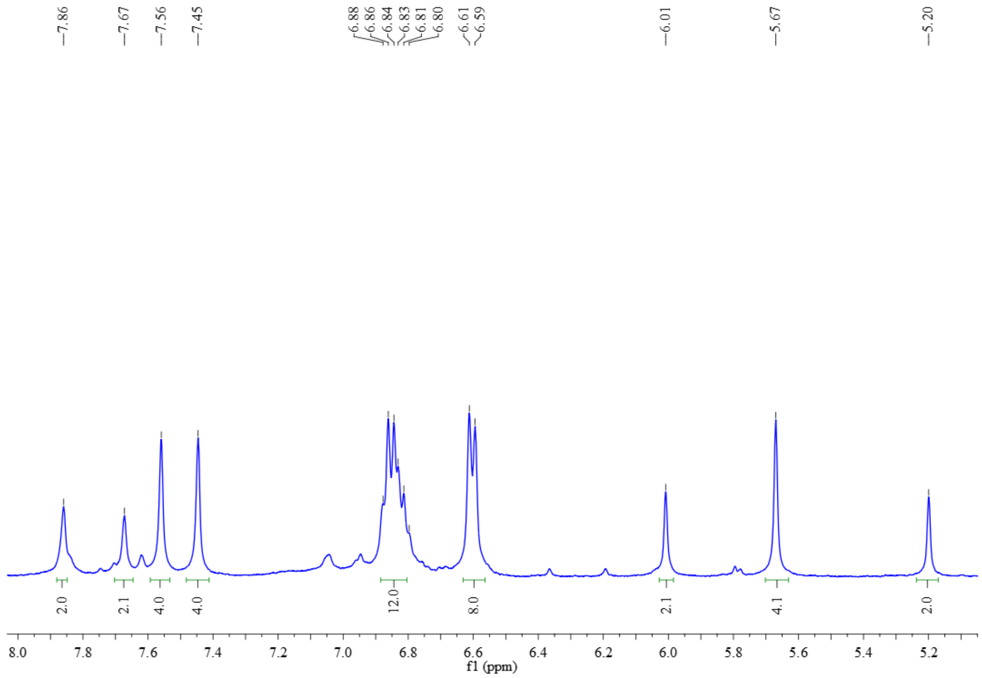

Figure S17. ^{1}H-NMR spectrum- DMSO-*d*$_6$ of tetra(phenyl)-pyrogallol[4]arene(**2b**)

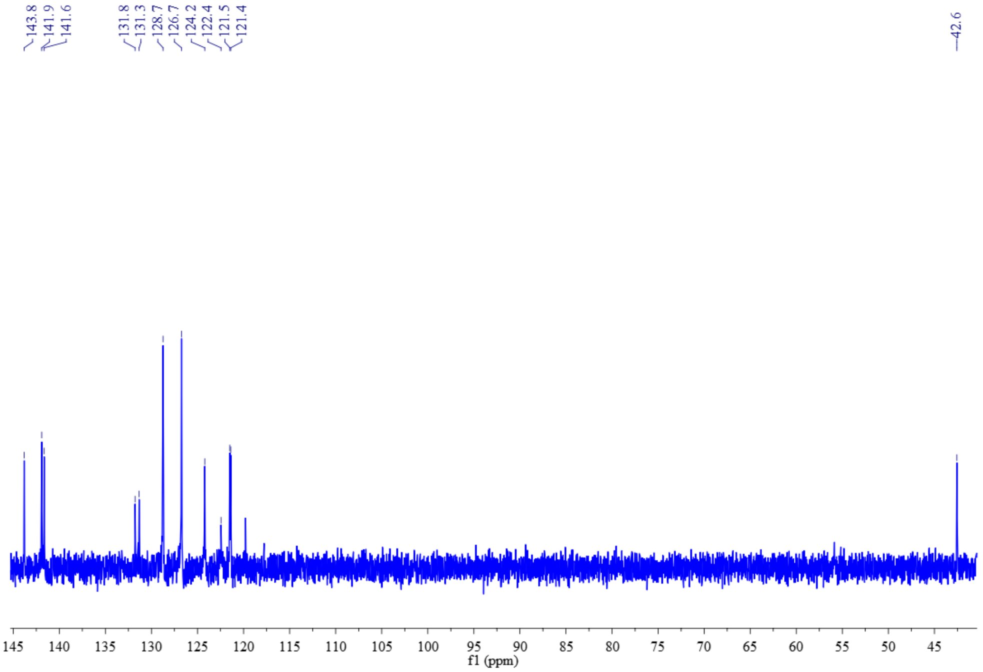

Figure S18. ^{13}C-NMR spectrum-DMSO-*d*$_6$ of tetra(phenyl)-pyrogallol[4]arene(**2b**)

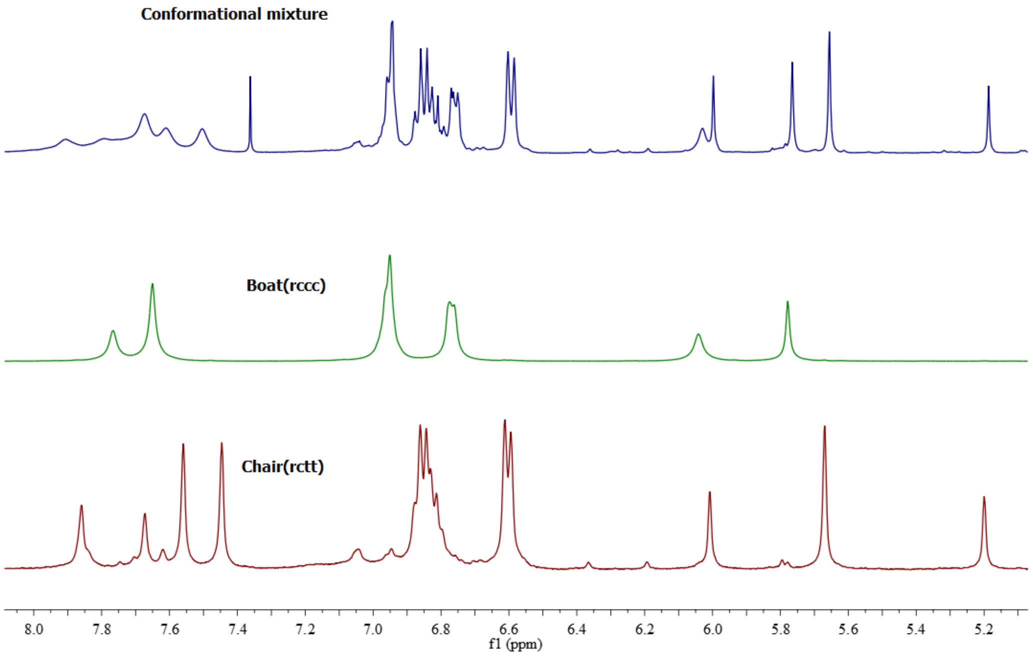

Figure S19. ^{1}H-NMR spectra of conformational mixture, *boat*(*rccc*)(**2a**) and *chair*(*rctt*)(**2b**) isomers
